# Dynamic relative regional strain visualized by electrical impedance tomography in patients suffering from COVID-19

**DOI:** 10.1007/s10877-021-00748-3

**Published:** 2021-08-13

**Authors:** Sven Pulletz, Lisa Krukewitt, Pablo Gonzales-Rios, Peter Teschendorf, Peter Kremeier, Andreas Waldmann, Amelie Zitzmann, Fabian Müller-Graf, Cecilia Acosta, Gerado Tusman, Daniel A. Reuter, Stephan H. Böhm

**Affiliations:** 1 Department of Anesthesiology and Intensive Care Medicine, University Medical Center Rostock, Rostock, Germany; 2 Department of Anesthesiology and Intensive Care Medicine, Klinikum Osnabrück, Osnabrück, Germany; 3 Simulation Center for Clinical Ventilation, Karlsruhe, Germany; 4 Department of Anesthesiology, Hospital Privado de Comunidad, Mar de Plata, Argentina

**Keywords:** EIT, Electrical impedance tomography, COVID-19, Strain, Lung injury, Dynamic relative regional strain, DRRS

## Abstract

Respiratory failure due to SARS-CoV-2 may progress rapidly. During the course of COVID-19, patients develop an increased respiratory drive, which may induce high mechanical strain a known risk factor for Patient Self-Inflicted Lung Injury (P-SILI). We developed a novel Electrical Impedance Tomography-based approach to visualize the Dynamic Relative Regional Strain (DRRS) in SARS-CoV-2 positive patients and compared these findings with measurements in lung healthy volunteers. DRRS was defined as the ratio of tidal impedance changes and end-expiratory lung impedance within each pixel of the lung region. DRRS values of the ten patients were considerably higher than those of the ten healthy volunteers. On repeated examination, patterns, magnitude and frequency distribution of DRRS were reproducible and in line with the clinical course of the patients. Lung ultrasound scores correlated with the number of pixels showing DRRS values above the derived threshold. Using Electrical Impedance Tomography we were able to generate, for the first time, images of DRRS which might indicate P-SILI in patients suffering from COVID-19.

*Trial Registration* This observational study was registered 06.04.2020 in German Clinical Trials Register (DRKS00021276).

## Introduction

Towards the end of 2019 the novel Corona virus SARS-CoV-2 started spreading from China across international borders causing a pandemic with the new contagious disease, named COVID-19 [1]. Respiratory failure due to COVID-19 pneumonia may progress rapidly to an ARDS-like clinical picture with an extremely inhomogeneously damaged lung [2]. CT scans show a lung morphology ranging from focal unilateral to diffuse bilateral ground-glass opacities with or without consolidations [3]. These patients often develop, even in the early phase, a severe hypoxemia [4] and a pathologic respiratory drive with high respiratory load. During the course of the disease this increased respiratory drive, which induces high tidal strain and energy loads on the vulnerable lung tissue, increases the risk for Patient Self-Inflicted Lung Injury (P-SILI) [5]. Data from the ROSE trial in patients undergoing assisted spontaneous breathing suggest that high levels of positive end-expiratory pressure (PEEP) might reduce the risk of P-SILI [6]. To date, very little is known about the impact of the high respiratory drive or P-SILI on the fragile lung tissue in COVID-19 patients [7]. It is expected that in COVID-19 lungs the same size of tidal volume will meet a significantly smaller functional lung size and an inhomogeneously distributed lung damage [8].

We postulate that the above inhomogeneous regional behavior of the lung characterized by areas of overdistension adjacent to areas of atelectasis can best be described by Gattinoni’s concept of strain, which is defined as the changes in the lung tissue induced by inspiration in relation to its relaxing state [9]. Thus, during spontaneous breathing, global strain can be derived from the ratio of tidal volume (VT) divided by functional residual capacity (FRC).

While this concept has been proven on a global level for the entire lung [10], the regional distribution of such strain within the lungs was demonstrated in computer-tomographic (CT) studies in experimental animals [11], and confirmed in a pilot study in lung-healthy patients [12]. These findings have recently been taken a step further by combining CT with electrical impedance tomography (EIT) in a proof-of-concept study in ARDS patients [13].

While these results under experimental conditions are encouraging these approaches do not meet the requirements for a monitoring modality capable of visualizing the non-homogenous distribution of ventilation within the COVID-19 lungs. An ideal solution would reveal in real time the risk for P-SILI or its damaging local effects.

**Fig. 1:**
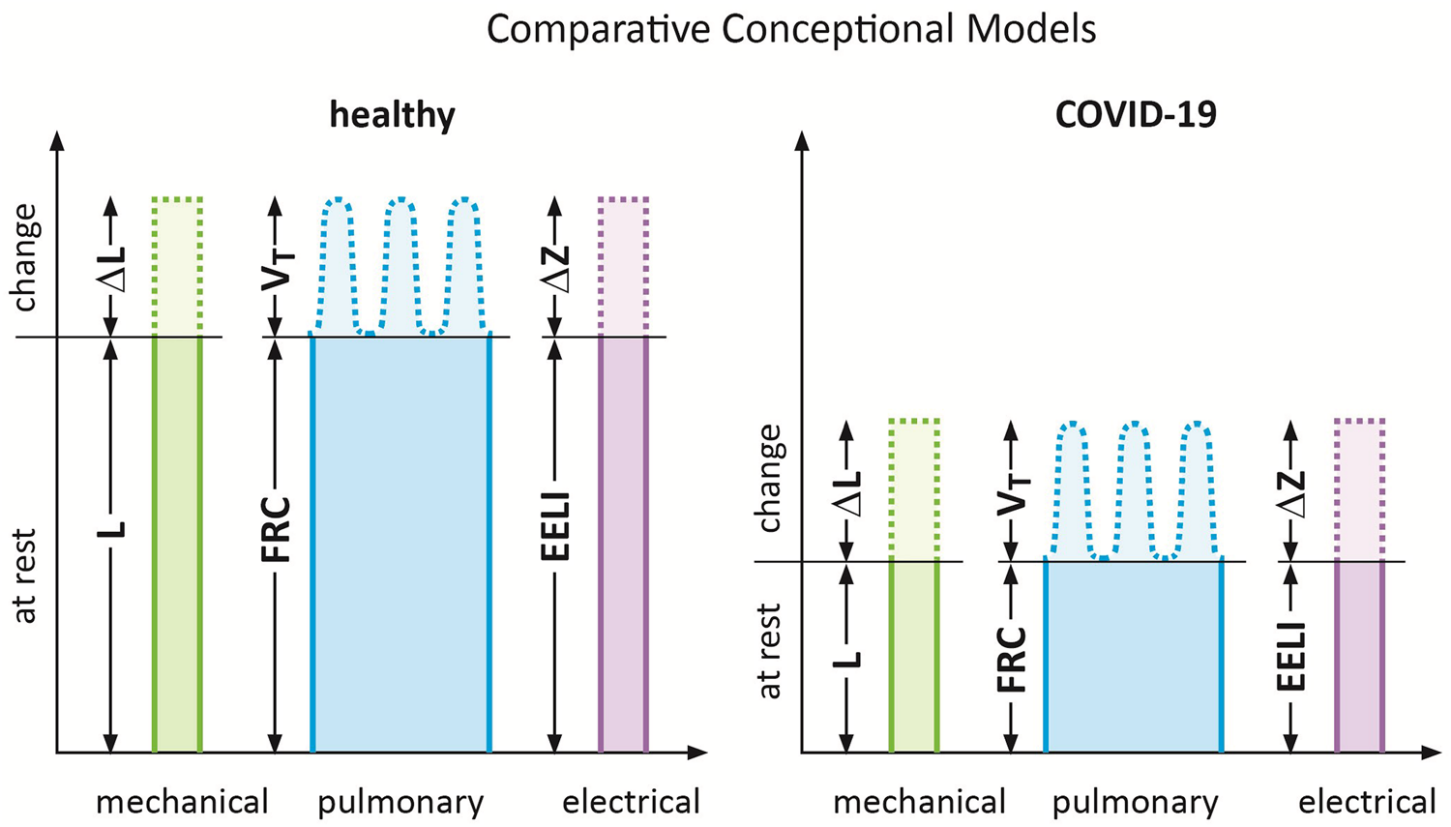
Conceptional drawing depicting mechanical, pulmonary and electrical models of strain in healthy and COVID-19 lungs. Strain is defined as the ratio of elongation (∆L) or tidal volume (VT) divided by the resting length (L) or volume (FRC), respectively, which in electrical impedance tomography are reflected by the changes in impedance (∆Z) in relation to the impedance at rest (Z)

EIT is a non-invasive bedside tomographic functional imaging technology [14]. It requires an electrode belt placed around the patient’s chest to apply alternating unnoticeable electrical currents in the low mA range. These electrical currents pass through the chest creating low voltages at the electrodes, from which up to 100 tomographic images per second are reconstructed. These high time-resolution images reflect the regional electrical properties within the lung tissue, which are influenced by the respiratory and cardiac cycles. In contrast to CT scans EIT-images do not display morphological but functional information showing regional tidal ventilation, local lung recruitment, expiratory time constants and the distribution of lung perfusion to name but a few. EIT also measures end-expiratory lung impedance (EELI), a parameter which correlates with FRC not only at the global but also on a regional or pixel level [14]. This impedance at end expiration has a significant influence on the breath-induced changes in the electrical properties to be presented in this study (Fig. 1).

Therefore, we introduce in this current pilot study a novel kind of functional EIT-image representing the mechanical strain inflicted on the inhomogeneous lung tissue by the spontaneous breathing efforts of SARS-CoV-2 positive patients. We hypothesize that patients with pneumonia due to COVID-19 will show both, higher global and DRRS values than the lungs of healthy volunteers.

## Methods

After ethical approval of the local ethics committee of the University Medical Center Rostock (A2020-0072) and written informed consent we included ten patients with COVID-19 pneumonia and ten healthy volunteers. This observational study was registered in German Clinical Trials Register (DRKS00021276).

Patients with no preexisting chronic lung disease but a positive test for SARS-CoV-2 and a clinically relevant pneumonia needing hospitalization were included. All patients received flows of oxygen up to 5 L/min via nasal cannula or High Flow Oxygen Therapy according to institutional standards. Subjects with an age less than 18 years, hemodynamic instability, history of severe chronic obstructive pulmonary disease, pregnancy, and contraindications for the use of EIT (e.g., presence of pacemaker, open chest wounds or respective surgical wound dressing in the belt area) were excluded from the study.

### Study protocol

Upon inclusion, oxygen demand (L/min) and both, peripheral pulse oximetry saturation and capillary blood gas samples were gathered. All EIT measurements were performed in the sitting, supine, left and right lateral position. Volunteers were examined only once in all of the above body positions, patients a second time three days apart undergoing additional ultrasound examinations in these positions and at both points in time. Only supine data are presented in this paper. During and after each measurement procedure the patient’s clinical status was assessed by the investigators and a subjective impression was documented.

### EIT measurements

The thorax circumference of each patient was measured, and a textile electrode belt of appropriate size placed around the chest along the 6th intercostal space. During each examination period electrical impedance tomography data of at least 10 consecutive breaths were recorded at a sampling-rate of 48 Hz by the Sentec BB^2^ (Sentec AG, EIT branch, Landquart, Switzerland) [15]. From these measured voltages patient-specific ventilation images of breathing-induced impedance changes were calculated in relation to a reference measurement using the manufacturer’s imaging algorithm.

### DRRS images

**Fig. 2:**
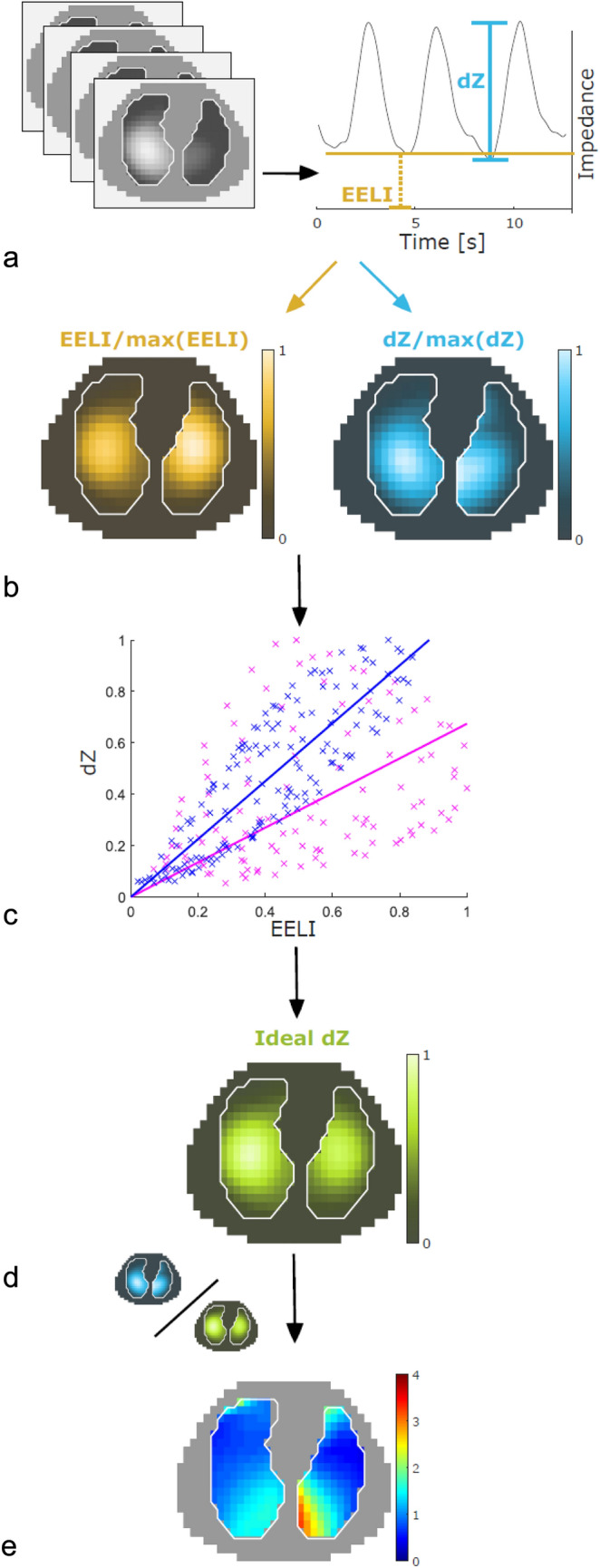
Method for defining Dynamic Relative Regional Strain (DRRS) described with exemplary images from one COVID-19 patient: **a** A series of EIT images is recorded at a sampling rate of 47.68 Hz. Only pixels from within the regions of the left and right lungs as defined by contours derived from CT images are considered. Breath-wise impedance change and mean end-expiratory impedance are calculated for each pixel. **b** Normalized distributions for dZ and EELI are calculated. **c** For each pixel, dZ is plotted against EELI. A linear regression is used to determine a straight line of constant lung strain assumed to be physiological for each of the lungs (magenta: left lung, blue: right lung). **d** An image of ideal dZ is calculated from the linear regression and the measured EELI values. **e** Finally, an image of DRRS is generated by dividing the measured dZ by the reference image dZ ideal generated from the linear regression

DRRS was calculated offline using Matlab R2081b (The MathWorks, Natick, Massachusetts) taking only pixels from within the lung regions into account. We identified the regions of interest (ROI) within the chest contours of the right and left lung based on a three-dimensional thoracic model. This model was created from computed tomography (CT) scans [16]. Knowing that absolute tidal volumes in milliliters cannot be obtained by EIT without prior calibration [17], it has been shown that impedance change (dZ) increases linearly with change in lung volume (dV) [18, 19]. Thus, EIT pixel values should reflect impedance changes that are proportional to the regional change in volume during tidal ventilation. In addition, Reinartz et al. and other groups showed that end-expiratory lung impedance increased linearly with end-expiratory lung volume [20–22]. Based on the above proportionalities it is further assumed that in healthy lungs the ratio of dZ to EELI is constant, also. Therefore, after determining breath-wise dZ and EELI values for each pixel, normalized distributions for dZ and EELI were calculated and dZ plotted against EELI. Based on the data points of each lung two regression lines representing the physiological DRRS within each one of the lungs were plotted. From these regressions, for each pixel and EELI value an ideal dZ was calculated and an image showing the distribution of ideal dZ was generated. The term ideal dZ was chosen to indicate the ideal matching of EELI and dZ reflected by the regression line representing the behavior of healthy lung tissue in both, healthy and sick lungs. The term ideal although reflects the therapeutic goal of maintaining the ideal relationship between tidal volumes and end-expiratory lung volumes by avoiding excessive PEEP-induced lung volumes (static strain) or excessive tidal volumes (dynamic strain) [9, 23]. Finally, the normalized dZ image was divided by the ideal dZ image, the result of which is an image of DRRS. The calculation of DRRS is schematic shown in Fig. 2.

### Histograms

To graphically represent the frequency distribution of DRRS values histograms were plotted for each image. As a result of the linear regression used to obtain the reference image dZ_ideal, DRRS values in the histograms around 1.0 should reflect the deformation of healthy lung tissue during normal breathing, therefore a cut-off for pathologic values was determined.

### Lung ultrasound

A standardized lung ultrasound (LUS) examination in six fields per hemithorax (upper/lower chest areas at parasternal, anterior axillary and posterior axillary line) was performed in all patients using a convex ultrasound probe (2–5 MHz, penetration depth 12 cm) of the Sparq ultrasound machine (Philips Healthcare, Eindhoven, Netherlands). In the supine position, the probe was placed longitudinally as recommended by expert consensus [24]. Normally aerated lung tissue was identified by lung sliding and A-lines while subpleural consolidations appeared as atelectasis in association with the following signs: absence of lung sliding, presence of multiple spaced apart B-lines or coalescent B-lines born in subpleural consolidation.

We determined the LUS aeration score of each lung field separately based on the following 4 standardized patterns: Score 0: predominant A-lines or < 3 separated B-lines. Score 1: at least three B-lines or coalescent B-lines occupying < 50% of the screen. Score 2: coalescent B-lines occupying > 50% of the screen. Score 3: large consolidations (at least > 1 cm). From the scores of all fields a final total score for each examination was obtained by summing up the scores of 12 areas examined [25]. Image evaluation and scoring were performed independently by two blinded LUS experts (GT and CA) not involved in the care of these patients.

### Statistics

All statistical analyses were performed using SigmaPlot 12.0 (Systat Software, Inc., San Jose, California, USA). All data were tested for normal distribution. A t-test for unpaired measures was used to test for differences between patients and healthy volunteers. A t-test for paired measures was used to compare the patients’ initial measurement with the subsequent one.

The number of pixels exceeding the threshold were counted so that the mean, standard deviation and a cumulative value could be derived. Regression analysis for the number of pixels with DRRS values above the threshold and the LUS findings was performed by a least square method and the correlation coefficient calculated. Differences were considered statistically significant at p < 0.05.

## Results

Patient demographics are shown in Table 1. EIT and LUS data were of sufficiently high quality in all subjects to warrant further analysis. All patients suffered from COVID-19 pneumonia with increased breathing effort, all of them requiring supplemental oxygen with flows between 2 and 4 L/min via nasal cannula resulting in SaO_2_ of 94 ± 2% and PcCO_2_ 30 ± 5 mmHg. Only patient 9 was supported by HFOT. All volunteers were healthy with no history of lung disease.

**Table 1:** Demographics presented as mean ± standard deviation

Variable	Patients (n = 10)	Volunteers (n = 10)
Sex	4 female/6 male	4 female/6 male
Age (years)	55 ± 21	32 ± 8
Height (cm)	172 ± 6	178 ± 10
Bodyweight (kg)	79 ± 17	75 ± 17

**Fig. 3:**
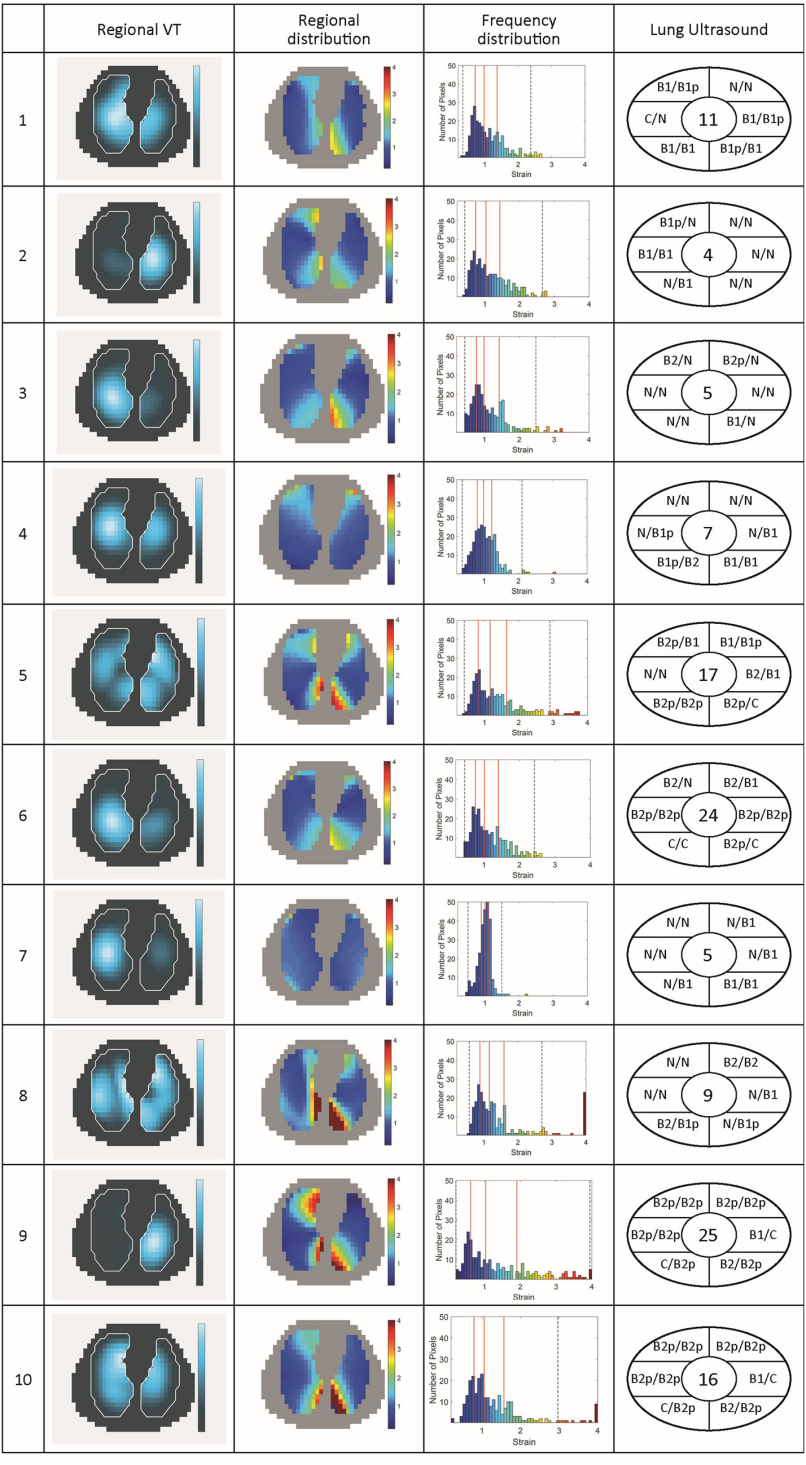
Regional distribution of ventilation and Dynamic Relative Regional Strain (DRRS) images of ten patients with COVID-19 pneumonia. DRRS is presented as a transversal functional image, in the form of a histogram showing its frequency distribution. Vertical solid lines within the histogram indicate median, 25% and 75% quartiles whereas dotted lines represent the 1st and 99th percentile. For each patient the results of the lung ultrasound examination in 12 lung fields are shown in the approximate location (upper/lower chest areas at parasternal, anterior axillary and posterior axillary line) within the schematic outline of the thorax. The quantitative total ultrasound score is depicted in the middle (Score 0: predominant A-lines or < 3 separated B-lines = N; Score 1: at least three B-lines or coalescent B-lines occupying < 50% of the screen. Score 2: coalescent B-lines occupying > 50% of the screen = B. Score 3: large consolidations = C). The letter “p” indicates the presence of an irregular pleural line or small consolidations (< 1 cm)

Figure 3 shows regional distribution of ventilation and DRRS images of all patients together with lung ultrasound scores.

**Fig. 4:**
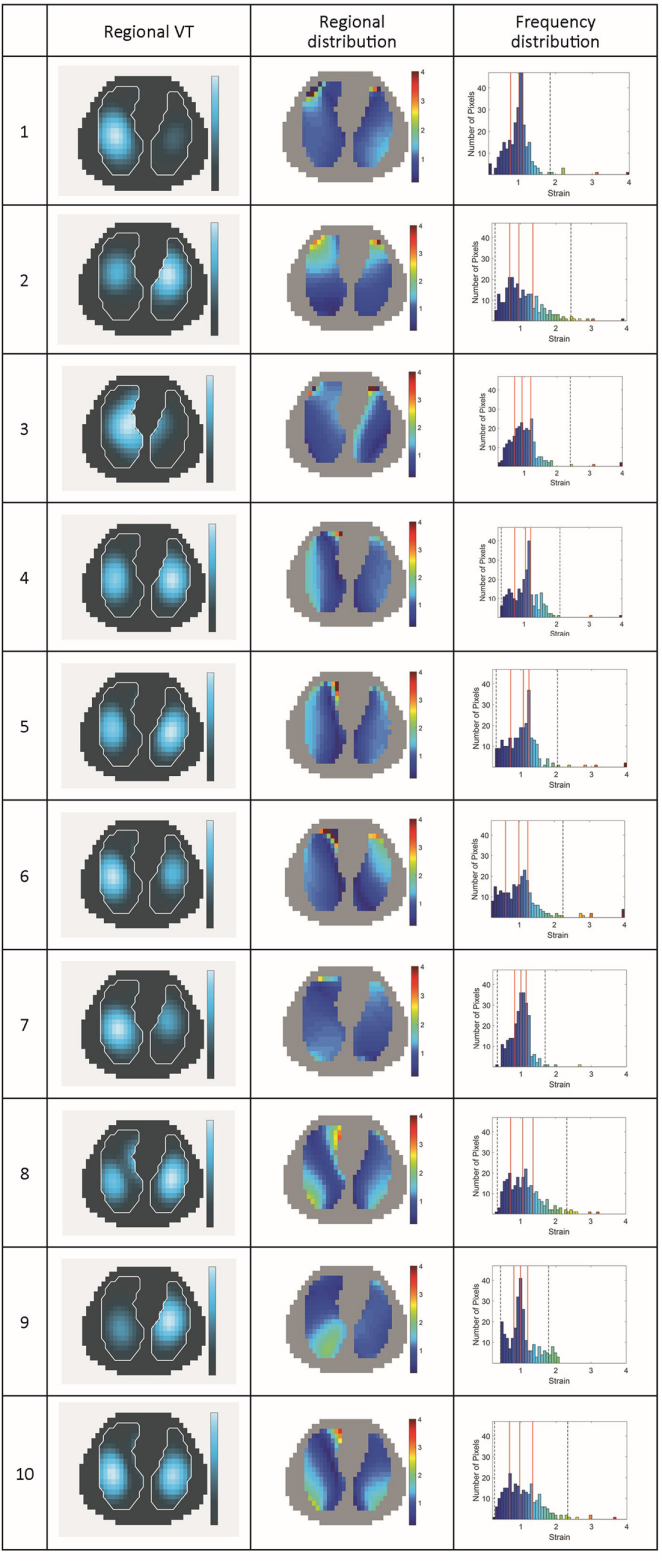
Regional distribution of ventilation and Dynamic Relative Regional Strain (DRRS) images of ten lung healthy volunteers. DRRS is presented as a transversal functional image and in the form of a histogram showing its frequency distribution. Vertical red lines indicate mean, 25% and 75% quartiles doted lines the 1st and, 99th percentile

Figure 4 presents regional distribution of ventilation and DRRS images of the volunteers. On visual inspection ventilation was more equally distributed between the right and left lung of volunteers than of patients.

The cut-off for pathologic DRRS value was determined to be 2.07 with 97.5% of all DRRS values of the healthy controls lying below this threshold. The mean and cumulative number of pixels with DRRS values above 2.07 were significantly higher in patients (23 ± 17 mean ± SD; n = 236) than in the control group (6 ± 4 mean ± SD; n = 63) and appeared mainly in the peripheral lung.

In patients subpleural atelectasis, B-lines and consolidations were the most frequent abnormal findings in the LUS images. There was a linear correlation between the total ultrasound score and the pathologic DRRS values (r = 0.57).

**Fig. 5:**
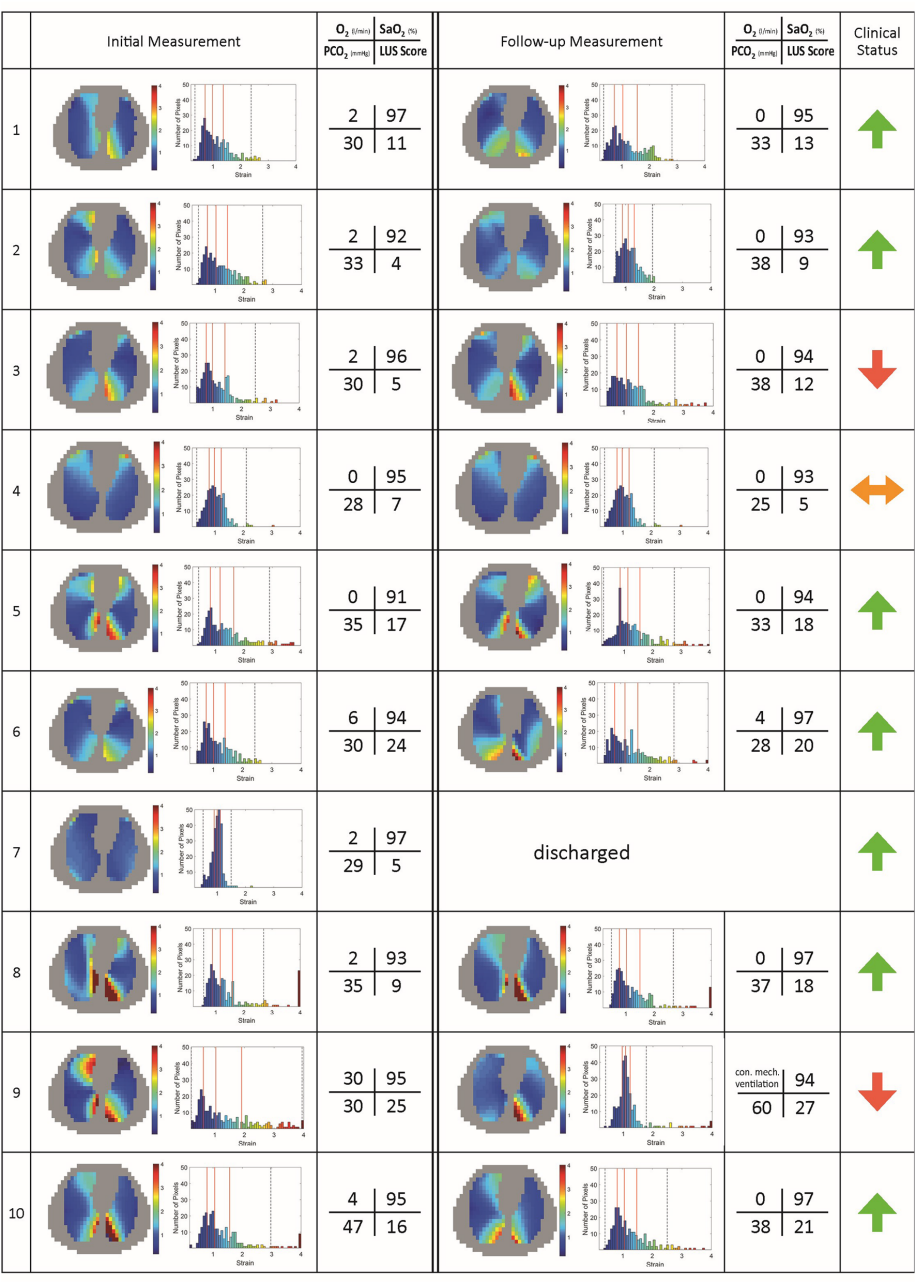
Dynamic Relative Regional Strain (DRRS) maps with frequency distributions, oxygen demand (L/min), pulse oximetric hemoglobin saturation (SpO_2_), PaCO_2_ and the total lung ultrasound score together with the clinical course indicated by arrows of the initial and the follow-up measurement three days later are presented

A follow-up measurement (Fig. 5) could not be performed in patient 7 who was discharged. During the initial examination, patient 9 was treated with high-flow-oxygen-therapy at 30 L/min. Two days later she became hypoxic requiring mechanical ventilation on the ICU with FiO_2_ 0.6, Pinsp 27 cm H_2_O and PEEP 15 cm H_2_O and died 10 day thereafter. All other patients recovered from COVID 19. DRRS patterns and locations comparable to the initial ones were found in all patients during the follow-up measurement with no statistical quantitative difference between them.

## Discussion

In this study we present for the first-time images of DRRS as a possible indicator of P-SILI in spontaneously breathing patients with SARS-CoV-2 pneumonia. Compared to healthy volunteers COVID-19 patients presented significantly more DRRS, which was distributed inhomogeneously. Elevated DRRS values indicate an inadequate relationship between the size of the tidal volume and the respective aerated volume of the affected lung region. The lung injury caused by SARS-CoV-2 may induce a vicious circle, in which the patient’s increased breathing effort may aggravate the patchy injury [5].

Thus, EIT has the potential to detect the incipient risk of severe lung damage due to DRRS, even before the subjects need respiratory support. It seems possible that the DRRS in these spontaneous breathing patients with high respiratory drive might be a relevant factor for P-SILI in COVID-19 patients.

While COVID-19 patients show an inhomogeneous distribution of ventilation and perfusion [26] compared to healthy volunteers, this ventilation pattern alone does not allow any inferences on the distribution of DRRS. This was expected since the size of the regional tidal ventilation as displayed in the ventilation images does not tell anything about the size of regional aerated lung volume it is delivered to. Thus, according to Amato et al., the DRRS images should contain aggregate information about both, the regional distribution of the tidal breathing as well as the respective size of the functional lung tissue (functional lung size) [10].

Breathing causes a physical deformation even of the healthy lungs, which will cause some level of DRRS. However, DRRS is expected to be low and distributed over a narrow range as the results of our healthy volunteers confirm. As a consequence of the normalization procedure the mean DRRS value was 1 with normal values ranging from 0 to 2 in a kind of Gaussian type of frequency distribution. DRRS values higher than 2 seem to reflect pathologies caused either by the disease itself, P-SILI or both. Our sequential measurements showed that high and increasing DRRS values appear to be indicators of increasing lung injury while decreasing lower DRRS seem to indicate the recovery of patients.

Furthermore, the sequential measurements taken three days apart reveal that the location and pattern of the DRRS distribution within the lungs were reproducible despite the removal and reapplication of the EIT belt. The individual correlations for both lungs may have contributed to this apparent robustness. These findings confirm the reliability of EIT shown in previous studies [27] Therefore, EIT in general and the parameter DRRS in particular may offer the potential as monitoring tool for regional strain during the course of the disease.

Patient 9 was the only one needing ventilatory support with a PEEP of 15 cmH_2_O during the second examination. At this time, the histogram showed an overall reduction and narrowing of the DRRS values despite a decline of her clinical status. We speculate that this interesting finding might be explained by a PEEP-induced increase in end-expiratory lung volume while tidal volumes remained constantly low. However, no tidal volumes were measured during the initial examination under spontaneous breathing to compare with. Therefore, it cannot be ruled out that the small VT as part of the lung protective ventilation strategy applied in this patient might have caused this effect alone or in conjunction with the high PEEP. We are thus aware that during lung protective ventilation strain cannot be interpreted correctly without accounting for changes in the size of the functional lung size or the tidal volumes used [28].

Following the logic of mechanical ventilation with PEEP this ratio has to be changed dividing VT by end-expiratory lung volume (EELV). However, Chiumello et al. believe that in the latter case the volume above FRC gained by and attributed to PEEP should be added to the VT in the numerator changing the equation to read as follows: VT + V_PEEP_/FRC [9, 23, 28]. It is however questionable whether this gain in lung volume should be considered a strain enhancer or rather a factor decreasing the strain [29]. In the context of our study, however, this academic debate is irrelevant as all subjects were breathing spontaneously at ambient pressure resulting in an end-expiratory volume which per definition is nothing else but true FRC.

The results of this pioneering study of DRRS has hinted towards the clinical potential of strain to be an independent indicator of lung injury. However, further studies are needed to investigate the reproducibility of our findings and how strain is influenced by the disease process and its respective therapeutic measures such as respiratory maneuvers, posture changes, VT and PEEP as well as medications.

## Limitations

Although EIT as a technology has been proven multiple times as an reliable means of obtaining regional changes in lung volume [14, 19, 30] the novel parameter of DRRS we proposed here could only be validated indirectly by comparable measurements in healthy volunteers. Unfortunately, there is no imaging technology available, which could have served as an appropriate reference for DRRS without subjecting the patients to undue harm.

It is likely that the calculation of DRRS is vulnerable to motion artefacts, regional shifts of lung areas and to failing electrode contacts.

Knowing that LUS examination does not reach deep into the lung but reflects pathologies near the lung borders and that the standard fields of LUS used in this study overlapped only partially with the transversal plane of the preceding EIT examinations, we still used this technology to provide at least some evidence for the degree of lung injury. The correlation of the LUS score with the number of high DRRS values supports this approach.

Further clinical studies are needed to prove the clinical relevance of the parameter strain in particular DRRS.

Finally, our method requires that a sufficient amount of functionally normal lung tissue within each one of the two lungs is available to determine a robust linear correlation between dZ and EELI. We are certain that this requirement was met in our spontaneously breathing COVID-19-patients. However, it is possible that this may not be true in severely injured or massively collapsed lungs. Therefore, it remains to be determined whether our method will work reliably also under these circumstances.

## Conclusions

Non-invasive bedside measurement of DRRS by electrical impedance tomography in healthy and injured lungs was feasible. High DRRS values and their reproducible inhomogeneous distribution were characteristic for the lungs of COVID-19 patients. The imaging patterns we found could be indicators of incipient P-SILI.

## Data Availability

After publication data and material will be available.

## Code availability

The software code is patented. However, it will be integrated in an available EIT device.
